# Infant Circumcision for Sexually Transmitted Infection Risk Reduction Globally

**DOI:** 10.9745/GHSP-D-21-00811

**Published:** 2022-08-30

**Authors:** Brian J. Morris, Stephen Moreton, John N. Krieger, Jeffrey D. Klausner

**Affiliations:** a School of Medical Sciences, University of Sydney, Sydney, Australia.; b CircFacts, Warrington, United Kingdom.; c Department of Urology, University of Washington School of Medicine, Seattle, WA, USA.; d Department of Medicine, Population and Public Health Sciences, Keck School of Medicine of the University of Southern California, Los Angeles, CA, USA.

## Abstract

Population-based studies in high-income countries have failed to find that male circumcision protects against sexually transmitted infections. Using evidence from several sources, we show that male circumcision does protect against HIV during insertive intercourse for men who have sex with men.

## Key Messages

- Population-based studies in high-income countries have generally failed to find protection of male circumcision (MC) against sexually transmitted infections (STIs). This stems from the overrepresentation of men who have sex with men (MSM) in national STI statistics, with inability of MC to protect against STIs during receptive anal intercourse.
- Studies of men from settings where MC is performed early in life showed lower prevalence of STIs in circumcised men.
- However, a Danish database study reported an association of early MC with an increased risk of STIs. The study’s findings were underpowered, contained questionable statistical methods, failed to consider receptive anal intercourse by MSM, and failed to adequately exclude non-ethnic Danes.
- Biological plausibility of infant MC in reducing infection risk is supported by its well-established ability to protect against infant urinary tract infections, as well as pathogenic bacteria and other microorganisms, across all ages. Childhood MC appears effective for protection against various specific STIs irrespective of the country where it was performed.

## MALE CIRCUMCISION FOR HIV AND STI PREVENTION IN MEN

We review the evidence concerning whether or not male circumcision (MC) performed early in life reduces the risk of HIV and other sexually transmitted infections (STIs). We first present findings for men, and then for children. In so doing, we critically evaluate 2 recent studies in which data appeared to contradict the premise of MC being protective. Our commentary has important policy implications for the performance of infant MC globally.

High-quality research on male circumcision (MC) shows varying degrees of protection against a number of heterosexually acquired sexually transmitted infections (STIs)^1^^,^^2^ including HIV,^3^^,^^4^ oncogenic human papillomavirus (HPV) types,^5^^–^^12^ herpes simplex virus type 2,^13^^–^^16^ genital ulcer disease,^17^^,^^18^ syphilis,^19^^–^^21^ *Trichomonas vaginalis*,^22^ *Mycoplasma genitalium*,^23^ and chancroid.^19^ However, MC does not protect against low-risk HPV genotypes that cause anogenital warts because these infect the anogenital region more broadly (i.e., MC is less effective).^24^ Nor does MC protect against urogenital urethritis caused by *Chlamydia trachomatis* or *Neisseria gonorrhoeae*.^25^^–^^27^ We use the term STIs to in most cases refer to those that MC protects against (Table 1).^28^^–^^34^

**TABLE 1:** STIs That Male Circumcision Protects Against

	Decrease in Risk, %^a^ (95% CI)	Proportion Affected, %^b^	Study Type (Reference)	Number of Cases^c^^,^^d^^,^^e^
HIV (acquired heterosexually)	72 (48, 70)	0.1	Meta^3^	2,000
	60 (39, 80)		OS^4^	
High-risk HPV infection	57 (42, 77)	10	Meta^11^	160,000
Herpes simplex virus type 2	28 (8, 44)	4	RCT^29^	6,000
	34 (-12, 61)		RCT^30^	
Genital ulcer disease	48 (ND)^f^	1	OS^17^	16,000
	48 (27, 63)		OS^18^	
Syphilis	33 (17, 46)	1	Meta^19^	16,000
	42 (9, 63)		OS^20^	
*Trichomonas vaginalis*	53 (8, 75)	1	RCT^22^	16,000
*Mycoplasma genitalium*	46 (1, 71)	0.5	RCT^23^	8,000
Totals	–	17	–	218,000

Abbreviations: CI, confidence interval; HPV, human papillomavirus; MC, male circumcision; Meta, meta-analysis; ND, not done; OS, original study; RCT, randomized controlled trial; STI, sexually transmitted infection.

a Based on data for circumcised versus uncircumcised males.^31^^–^^34^

b The percentage of males who will be affected over their lifetime as a result of the single risk factor of retention of the foreskin. Data for STIs were estimated after taking into account the external factor of heterosexual exposure, and population prevalence of each STI in the United States, the United Kingdom, Canada, Australia, or other countries. Risk reduction conferred by circumcision was based mostly on data for the United States because more data were available for that developed Anglophone country.

Percent affected = fraction of males affected over their lifetime x risk reduction afforded by MC x 100. The more prevalent a condition, the greater the number of males that would be affected over their lifetime.

c Number of additional cases in the United States if NMC prevalence decreased from 90% to 10% was obtained by multiplying the fraction affected (from column 3) by 1,967,458 x (90–10)/100 = 1,573,966.

d Based on the latest U.S. Centers for Disease Control and Prevention Health Statistics data of 1,967,458 male births in 2019.

e If prevalence of MC performed predominantly in infancy were to decrease from approximately 90%, as seen in non-Hispanic white men in the United States,^28^ to a prevalence of approximately 10%, as seen in the United Kingdom and Europe.

f Instead, Nasio et al. state *P*<.01, to indicate statistical significance.

Much of the high-quality data on MC for STI prevention originated from large HIV RCTs involving mostly heterosexual men* in South Africa, Kenya, and Uganda.^35^^–^^37^ In South Africa, Kenya, and Uganda, most STIs involve heterosexual exposure. The strong protective effect of MC against HIV infection led the World Health Organization and the Joint United Nations Programme on HIV/AIDS,^38^ supported by funding from the U.S. President’s Emergency Plan for AIDS Relief, United States Agency for International Development, and the Bill and Melinda Gates Foundation, to roll out voluntary medical MC programs across 15 priority sub-Saharan African countries, which, accompanied by other measures, has reduced HIV incidence substantially.^39^ Major medical bodies and some MC opponents now accept that MC reduces HIV infection and perhaps other STIs in men in sub-Saharan Africa. However, some continue to question whether MC in infancy and childhood offers similar protection and whether the findings in sub-Saharan Africa apply to countries with higher socioeconomic status.

Evidence-based reviews by the American Academy of Pediatrics^40^^,^^41^ and the U.S. Centers for Disease Control and Prevention^42^^,^^43^ led to recommendations favoring MC in infancy and childhood to reduce the risk of HIV, other STIs, and an array of additional medical conditions in the United States.

The most recent meta-analysis found MC provides 72% protection against heterosexually acquired HIV infection.^3^ Because most of the current data are based on findings in sub-Saharan Africa, some have argued that these may not apply to high-income countries (HICs). To support this premise, it has been pointed out that MC is not associated with overall HIV prevalence in those HIC settings. This was apparent in recent Canadian studies.^44^^,^^45^

Because most of the current data on protection of MC against STIs are based on findings in sub-Saharan Africa, some have argued that these data may not apply to high-income countries.

A key difference between data from sub-Saharan Africa and HICs stems from the extent of particular sexual behaviors, notably, receptive anal intercourse. Men in the sub-Saharan African RCTs were mostly heterosexual, whereas data from HICs come from a mixture of heterosexual men and men who have sex with men (MSM). Crucially, it should be obvious that MC would offer no protection against STIs among MSM who adopt the receptive role during anal intercourse. As a result, studies of HIV incidence and HIV prevalence in men overall in HICs have failed to find a protective effect of MC against HIV infection.^46^^–^^48^ Most STIs transmitted to receptive MSM would presumably be anorectal. Although the collection of anorectal swabs for testing and diagnosing STIs was not performed in all studies, for HIV (and certain other STIs), diagnosis is done instead by seropositivity. The most recent meta-analysis^48^ identified studies in Sydney, Australia,^49^ and Buenos Aires, Argentina,^50^ with HIV data for both receptive and insertive MSM. Each study found that MC protected against HIV infection in MSM who practiced insertive anal intercourse. The study in Sydney involved 1,426 initially HIV-negative MSM, one-third of whom preferred the insertive role during unprotected anal intercourse.^49^ Of these insertive MSM, HIV seroconversions were seen in 2 of 279 circumcised MSM and 5 of 156 uncircumcised MSM (adjusted hazard ratio=0.11; 95% confidence interval [CI]=0.01, 0.92). In the Buenos Aires study of 500 MSM, HIV positivity was detected in 0 of 33 circumcised and 34 of 231 (14.8%) uncircumcised MSM (*P*=.02).

Failure to show a protective effect of MC against HIV in HICs may stem from not taking into consideration differences in risk for contrasting sexual practices among MSM. We illustrate this by an examination^51^ of the study findings by Nayan et al.^44^ Among 569,950 Canadian men from Ontario, HIV prevalence was only 2% (nonsignificantly) lower in the 203,588 (35.7% of the sample) who were circumcised (83% circumcised in infancy) compared with the 366,362 who were uncircumcised. Another recent Canadian study found that 14% of HIV cases in men occurred in heterosexuals, while 86% occurred in MSM.^45^ Since there were no data on the role taken during anal intercourse in these studies, we used data from the high-quality longitudinal study in Sydney,^49^ which, in contrast to Buenos Aires, is a setting more comparable to Ontario. The Sydney study found that during anal intercourse, 69% of MSM adopt the receptive role, for which MC offers no protection. It found that 33.1% of MSM practiced the insertive role; among these participants, 75.7% of HIV infections could be attributed to being uncircumcised. In contrast, among all participants, 8.7% of HIV infections could be attributed to being uncircumcised.

Failure to show a protective effect of MC against HIV in HICs may stem from not taking into consideration differences in risk for contrasting sexual practices among MSM.

A Cochrane meta-analysis has shown that circumcised insertive MSM are at 73% lower HIV risk.^47^ Using the Sydney data for percentage of MSM who are receptive^49^ and data for percentage of men who are MSM in Canada,^45^ 26.7% of HIV infections occur among insertive MSM. Adding 26.7% to the 14% of HIV infections found in heterosexual males in Canada,^45^ gives 40.7%, which when multiplied by 0.357 and then 0.73 gives 10.6%. And 10.6% of 203,588 gives 21,580, showing that only 3.8% of Canadian men are at lower HIV infection risk because they are circumcised. Factoring in the above, 15 of the 141 HIV cases in Nayan et al.^44^ were predicted to have occurred in insertive circumcised men and 126 cases in uncircumcised insertive men. A χ^2^ test comparing these numbers with the expected numbers (50 and 91, respectively) if circumcision had no effect, showed that circumcision reduced risk by ∼70% (χ^2^=38; *P*=7.2 x 10^–^ ^10^) (Figure).^51^ The critics’ calculations and conclusions^51^ were endorsed by Nayan et al.,^52^ the authors of the Ontario study that was evaluated.^44^

**FIGURE:**
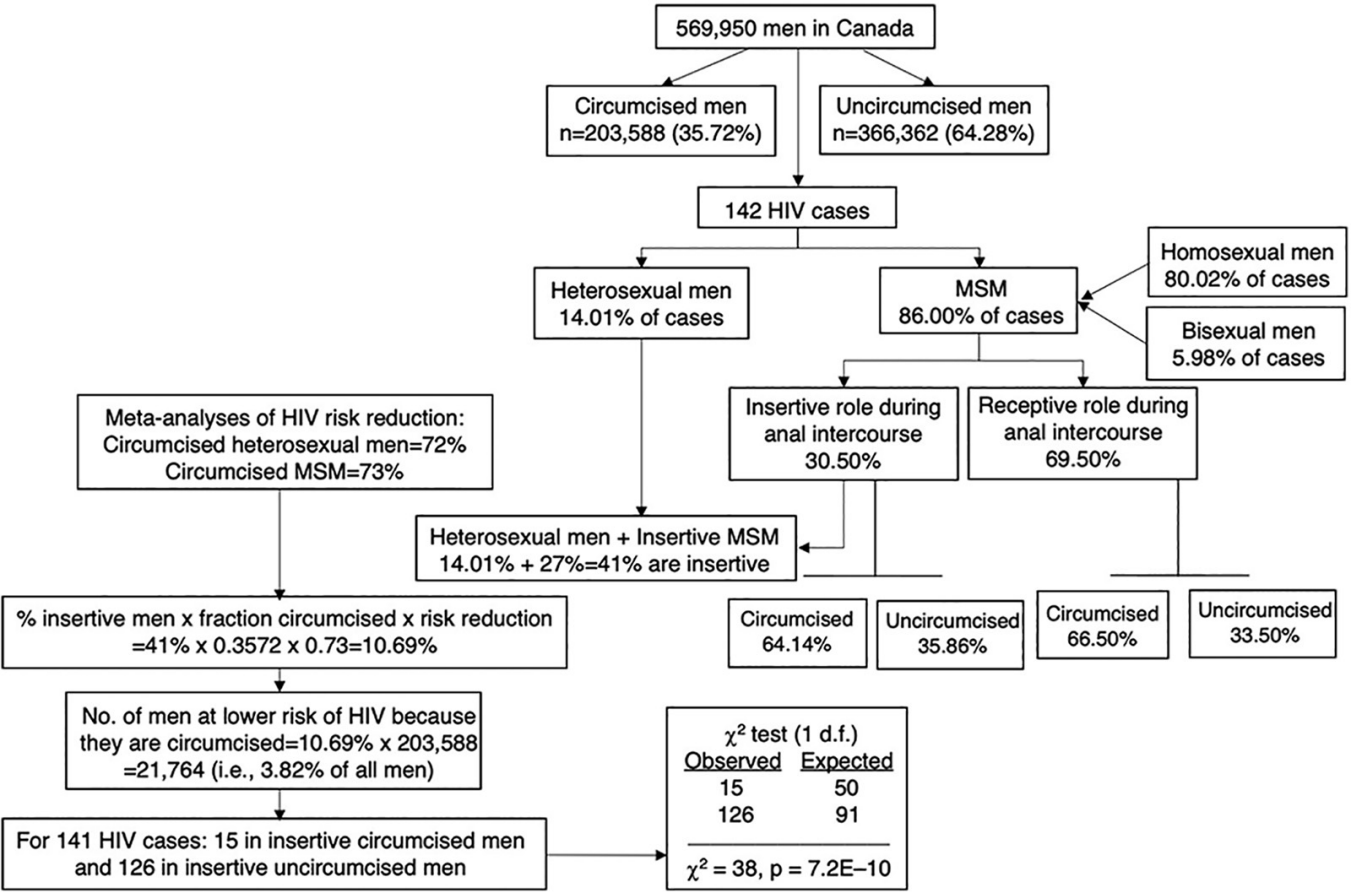
Flow Chart Showing How MC Was Strongly Associated With Reduced Risk of HIV Infection^51^^,^^a^ Abbreviations: MC, male circumcision; MSM, men who have sex with men. ^a^Inclusion criteria: English language, published after 1980, unique data on HIV in relation to circumcision status were examined. Despite null results for HIV and MC in a Canadian study,^44^ after allowing for sexual preference^45^ and percentage of MSM who are insertive and receptive during anal intercourse.

Among other STIs studied by the Sydney investigators, HPV prevalence was also found to be significantly lower in circumcised MSM who adopt the insertive role during anal intercourse.^53^ Intriguingly, high-risk HPV has been found in 9% of foreskin samples of prepubertal boys.^54^

Among women, a study of serodiscordant couples found those with a circumcised male partner exhibited a nonsignificantly 38% lower risk of HIV infection.^55^ Systematic reviews have documented consistent evidence for protection against HPV, cervical dysplasia, cervical cancer, HSV-2, *Trichomonas vaginalis*, syphilis, bacterial vaginosis, and chlamydia.^56^^,^^57^

### Studies That Included Males Circumcised in Childhood

All current RCT data on STIs and MC involve adult males in sub-Saharan Africa. It would be impractical and unethical to conduct an RCT on early childhood MC and acquisition of STIs later in life. Therefore, we searched for studies of STIs comparing men likely to have been circumcised early in life with uncircumcised men.^12^^,^^53^^,^^58^^–^^79^ Circumcised men in studies from HICs included mostly men who were circumcised as neonates but also some circumcised later in infancy or childhood (Table 2). In all of these men, STI risk was lower in circumcised compared with uncircumcised men. A contrary Danish study^80^ was not included in Table 2 and will be discussed in detail in the section analyzing contrary evidence. Table 2 also includes studies in low- and middle-income countries, all of which were on HIV. These found lower HIV prevalence among men circumcised in infancy and childhood.

**TABLE 2.** All Studies of STIs in Males Who Had Been Circumcised in Infancy or Childhood^a^^,^^b^

	Country	Reference
High-income countries		
HIV	USA	Warner et al. (2009)^58^
	Israel vs. Europe	Chemtob et al. (2015)^59^
HPV	USA	Baldwin et al. (2004),^60^ Daling et al. (2005),^61^ Nielson et al. (2007),^62^ Hernandez et al. (2008),^63^ (2010),^64^ Nielson et al. (2009),^65^ Lu et al. (2009),^66^ Guiliano et al. (2009),^67^ Han et al. (2017)^68^
	Australia	Poynten et al. (2012),^53^ Aung et al. (2018)^69^
	UK	Homfray et al. ( 2015)^12^
	France	Aynaud et al. (1999)^70^
HSV-1	USA	Van Wagoner et al. (2009)^71^
HSV-2, candidiasis, gonorrhea, and syphilis	Australia	Parker et al. (1983)^72^
Chlamydia, gonorrhea, genital warts, HSV-2, and nonspecific urethritis	New Zealand	Fergusson et al. (2006)^73^^,^^c^
Syphilis, gonorrhea, and anogenital warts	USA	Cook et al. (1994)^74^
Syphilis, chlamydia	USA	Diseker et al. (2000)^75^
Syphilis	Australia	Templeton et al. (2009)^76^
Low- and middle-income countries		
HIV	Algeria, Senegal,Gambia, Afghanistan,Bangladesh, Tajikistan, Pakistan, Iran	Addanki et al. (2015)^77^
	India	Reynolds et al. (2004),^78^Kenyon (2019)^79^

Abbreviations: HPV, human papillomavirus; HSV-1, herpes simplex virus 1; HSV-2, herpes simplex virus 2; MC, male circumcision; STI, sexually transmitted infection.

a Excluded was a Danish study by Frisch and Simonsen,^80^ which will be discussed extensively later.

b These found MC had a protective effect.

c This was a longitudinal birth cohort study.

In studies of men circumcised early in life, STI risk was lower in circumcised compared with uncircumcised men.

### Biological Plausibility

Biological plausibility is 1 of the 9 key criteria Bradford Hill describes as being required for causal inferences from observational studies.^81^ The strongest biological data suggest that the foreskin is highly susceptible to HIV infection.^82^^–^^87^ Inflammatory conditions and ulcerative STIs increase risk,^88^^–^^92^ as do coital injuries, to which uncircumcised men are prone.^93^^–^^95^ Risk is higher when foreskin surface area is large.^96^ Some protection against low levels of HIV is afforded by langerin, which is produced by the inner foreskin mucosal epithelium.^97^ However, langerin becomes overwhelmed at high HIV loads.^97^^,^^98^ Distinct lymphoid aggregates just beneath the basal membrane, which was densely populated with CD3^+^CD4^+^ T cells, and abundant superficial HIV target cells close to superficial blood and lymph vessels, provide possible anatomical explanations for the protective effect of MC against HIV infection.^84^^,^^99^ Breaches in the epithelial layer through trauma or inflammation, dendrites extending to the foreskin surface,^83^^,^^100^ and passive diffusion of HIV particles through normal foreskin epithelium, as well as through the more vulnerable underlying glans of uncircumcised men,^101^ may contribute to the susceptibility of an uncircumcised penis to infection.

But is foreskin retention in infancy a risk of infection by microorganisms? A common site of infection in infancy is the urinary tract. Strong data show that MC early in infancy protects against urinary tract infection. This type of infection in males is most common in infancy, affecting 1%–2% of uncircumcised boys compared to 0.1%–0.2% of boys who are circumcised.^102^^,^^103^ Protection, albeit progressively lower, extends over the lifespan.^102^ Swabs taken under the foreskin of boys aged 7 days to 11 years identified 50 bacterial isolates, most of which were multidrug-resistant strains.^104^ Colonization by pathogenic bacteria is much more prevalent under the foreskin of uncircumcised boys compared with the exposed glans region of circumcised boys.^105^^–^^116^ The foreskin is more prone to inflammation exacerbated by the predominantly anaerobic microbiome of the preputial space.^109^^,^^117^ Taken together, it seems biologically plausible for circumcision performed in infancy to also protect against infection of a healthy penis from HIV and other STIs later in life.

Some disregard the biology in arguing that data from African studies do not apply to HICs where sexual behaviors, such as the number of partners and condom use, may differ. Since the evidence we discuss indicates that the protective effect of MC is biological, this protection will still be present regardless of the number of partners or use of condoms.

## ANALYZING CONTRARY EVIDENCE TO THE PROTECTION OF MC AGAINST STIS

Given the strong data previously mentioned supporting the ability of MC to reduce the risk of STIs, the findings of a 2022 study by Frisch and Simonsen that reported a 53% higher STI risk among males circumcised at age 0–10 years (mean 5.9 months)^80^ appeared anomalous. Their study involved 810,719 males born in Denmark between 1977 and 2003 and followed up between January 1977 and November 2013 (27 years in total; on average 22 years). The oldest males would thus have been 27 years of age, and mean age of all males was 22 years. STI acquisition would have occurred mostly in the period of follow-up when the subjects were old enough to be sexually active. Parent-approved nontherapeutic MC is uncommon in Denmark. After excluding MC for foreskin-related medical problems and most Muslims, only 3,375 (0.42%) of the cohort were circumcised. Of 8 STIs evaluated, significant differences were found only for anogenital warts and syphilis. There were 74 cases of warts but only 4 cases of syphilis, as we will discuss.

We critically evaluated the findings of a 2022 Danish study that reported a 53% higher STI risk among males circumcised at age 0–10 years.

We conducted a thorough critical evaluation of this study’s design, cohort used, data analyses, and interpretation, as well as their failure to consider other factors, such as sexual preference. Our findings have substantial implications for global public health.

### Limitations in Cohort Used

#### Low Prevalence of HIV

Frisch and Simonsen found no HIV cases among 3,375 circumcised males in their study, compared with 321 among 807,344 uncircumcised males. They concluded that MC did not seem to reduce the risk of HIV infection, a conclusion that seemed to be based on a simulation study they performed in which they assumed similar rates of infection among circumcised and uncircumcised males in their study. Their conclusion is not consistent with their simulation. The simulation tested the null hypothesis of equal or higher rates of HIV acquisition in circumcised males against the alternative hypothesis that the rate is lower among circumcised males. In their results, they implied that rates of HIV infection among circumcised and intact males were not statistically significantly different.^80^

One might conclude that early MC could protect against HIV but that the limited number of cases was not sufficient to meet the conventional threshold of statistical significance. This conclusion is subject to the same caveat of low statistical power that applies to almost all STI analyses in their study.

#### Sexual Preference Not Considered

The database Frisch and Simonsen used did not record sexual preference. In Denmark, 3% of men may be homosexual and 4% bisexual.^118^ MSM are overrepresented in population STI statistics.^45^ Contrary to expectations, rather than the proportion decreasing in recent years owing to preexposure prophylaxis (PrEP), a meta-analysis has found that PrEP has been accompanied by increases in STI diagnoses, condomless sex, receptive anal intercourse, and sex with an HIV-positive partner.^119^ Thus, sexual orientation and role adopted during sexual activity represent potential confounding factors (see the calculations for the Canadian study^44^). Since most MSM receiving PrEP undergo regular anal STI screening as part of PrEP programs, such screening may help identify prevalent STIs, especially as most STIs are asymptomatic, so screening may substantially increase ascertainment. We presume that, as elsewhere, such screening includes testing rather than just self-reporting of symptoms.

Sexual orientation and role adopted during sexual activity represent potential confounding factors for risk of STIs.

#### Low Prevalence of MC

MC prevalence in Frisch and Simonsen’s study was only 0.42%. The extraordinarily low number of circumcised males in the study combined with a lack of consideration of sexual preferences among MSM may explain in part their failure to show a reduced risk of HIV and other STIs by childhood MC. Moreover, it would be naïve to assume that an equal proportion of circumcised and uncircumcised MSM practice receptive anal intercourse.

#### Low Prevalence of STIs

In Frisch and Simonsen’s study, the number of STI cases in circumcised males (besides anogenital warts, 74 cases), was very low: 2 of genital herpes, 4 of syphilis, 5 of gonorrhea, and 3 cases in total of chlamydia, granuloma inguinale, and trichomoniasis (presumably 1 case of each). The low number of events in the circumcision group meant that there was a high chance of Type II error (i.e., failure to reject the null hypothesis because statistical power is insufficient). Their regression analyses may thus suffer from overfitting and instability.

In a 2011 database study (of sexual function), Frisch et al. acknowledged instability in their statistical analyses because only 5% of the cohort used in that study was circumcised.^120^ Since the percentage of circumcised males in the 2022 study was very low (0.42%), instability as a factor in their analyses would weaken the reliability of the findings despite the possibility of their study being, as they claim, “by far the most powerful prospective study to date.”

The subjects were drawn from the Civil Registration System of Denmark using National Patient Register surgery codes 55620 for 1977–1995 or KKGV20 from 1996 onward. From 2004 Denmark started removing nontherapeutic MC from publicly subsidized records, thus resulting in incomplete records for circumcisions performed thereafter. But from the start of 2017 all circumcisions, at home or in a clinic, had to be registered.^121^ Codes for nontherapeutic circumcisions were used to exclude circumcisions for medical reasons, so contributing to the low proportion of circumcised males in their cohort. The effect of these various changes in procedures for recording circumcisions is not clear, nor was it addressed by Frisch and Simonsen.

### Limitations in Interpretation

A key flaw in the Frisch and Simonsen findings is the probability that some STI events (including the largest category, anogenital warts) were actually related to the sexual partners’ MC status rather than the subjects’ own status. Because the proportion of circumcised males in their cohort was only 0.42%, the likelihood of having a circumcised sexual partner was very low and was approximately equal for the 2 groups. For MSM, the likelihood of having an uncircumcised sexual partner was approximately 99.6%.

### Limitations of Hazard Ratios

Their use of the Cox proportional hazard model and their reporting of hazard ratios presents potential problems.^122^ Instead, Kaplan-Meier Survival Curves should have been used to describe and explore the relationship between the putative predictive factor (circumcision status) and the time to event (STI).^122^^,^^123^ Because a survival curve was not included, the reader cannot assess the validity of the assumption of proportional hazards. Frisch and Simonsen did not address this key assumption before selecting the Cox proportional hazards model. A Kaplan-Meier curve would assist the reader to judge whether the Cox model was appropriate.

- Their Cox regression model used a baseline timepoint of date of birth for subjects. This seems problematic because subjects are not susceptible to the event occurring until many years after birth when they become sexually active. Boys born late in the study period would not have been sexually active. It also means that their use of “person-years” of follow-up is quite misleading because the cohort is not at risk of the event for much of the follow-up period.
- The Cox model assumes that the event can occur only once to each subject. However, STI events often occur more than once, so a recurrent event model may be more appropriate.^124^ This could take the form of a generalized linear model with a Poisson or logistic link for the events as a dependent variable.

No data were provided for oncogenic HPV genotypes. These are present in 22%–45% of men in Denmark,^125^^,^^126^ where, as in other countries, lower prevalence has been reported in those men who are circumcised.^127^^,^^128^ Their study only reported data for anogenital warts, which are caused by low-risk HPV genotypes known to infect the entire anogenital region, rather than the distal penis where high-risk HPV infects and which has a prevalence in Denmark of up to 11%.^127^

Given the calculations above for HIV and sexual preference, the majority of the 74 cases of anogenital warts found in circumcised males could be in receptive MSM. Taken together, MC status should have no overall impact on risk of most anogenital warts.

### Failure to Exclude Ethnic and Cultural Minority Groups in Which MC Is Common

Frisch and Simonsen acknowledged that non-ethnic Danes, specifically Muslims, may have different sexual behaviors, so they sought to exclude them. Therefore, they excluded males whose parent or grandparent was born in 17 countries with a Muslim majority (Turkey, Iraq, Pakistan, Iran, Somalia, Lebanon, Afghanistan, Morocco, Egypt, Syria, Indonesia, Algeria, Jordan, Bangladesh, Kuwait, Tunisia, and Kosovo). While doing so may have helped reduce 1 element of confounding, it also reduced power. As a result, of the 810,719 supposed Danish-born non-Muslim males in their study, only 3,375 (0.42%) had undergone MC at ages 0–10 years. The fact that Denmark is a non-circumcising culture raises the question, what was the ethnicity of the 3,375 circumcised males? This is a key issue in considering unknown and unaccounted for confounding factors that might exist in their data.

Socioeconomic factors, discrimination, and distrust influence access to health care and the likelihood of contracting an STI. These affect minority groups disproportionately. As the U.S. Centers for Disease Control and Prevention explains on its website^129^:

*There are higher rates of STDs among some racial or ethnic minority groups compared to whites … these higher rates are not caused by ethnicity or heritage, but by social conditions that are more likely to affect minority groups. Factors such as poverty, large gaps between the rich and the poor, fewer jobs, and low education levels can make it more difficult for people to stay sexually healthy … In communities with higher STD rates, sexually active people may be more likely to get an STD because they have greater odds of selecting a partner who is infected.*

This is seen as well in European and British settings where ethnic minorities are often at greater risk for STIs.^130^^–^^132^

While it may be argued that second- or third-generation immigrants, as those included in Frisch and Simonsen’s cohort, may be assimilated into Danish ways, the extent of this is unquantifiable. They are also more likely to associate (and have sex) with new immigrants of the same ethnicity or visit their ancestral homelands where STIs may be more prevalent (as in the United States and sub-Saharan Africa) and contract an STI there, putting them at greater risk than their ethnic Danish peers. While Danish people may also travel abroad, the destination chosen would not be influenced by the factor of ethnic origin that is more likely to apply to ethnic minorities. Again, these effects cannot be quantified.

With ethnicity a source of confounding, in studies of sexual health the cohort should be ethnically homogenous unless ethnicity is being studied. The European Centre for Disease Prevention and Control recognizes the importance of collecting data on ethnicity in relation to HIV in Europe, observing that ethnic minorities in Europe are disproportionately affected by HIV.^133^

In studies of sexual health, the cohort should be ethnically homogenous unless ethnicity is being studied.

Frisch and Simonsen stated that after excluding subjects from the 17 Muslim countries, no other single country accounts for more than 0.1% of immigrants to Denmark. Thus, they did not exclude Jewish, American, African, and perhaps males born to other ethnicities, cultures, or races in which circumcision is commonly practiced. Statistics for immigrants in Denmark are available from 1980 onward at www.statbank.dk, and further data on the proportion of Muslims in various countries are available from other publications.^134^^–^^138^ The Statistics Denmark database^139^ documents an enormous increase in the number of Muslims in Denmark after 1996, when an influx of Bosnians began. However, most of the children born that recently would not have been sexually active during Frisch and Simonsen’s study period ending in 2013.

Therefore, we extracted data for the year 1980 (Table 3), close to the start of the period used by Frisch and Simonsen, even though this would have resulted in an underestimate, because immigration increased throughout the study period.

**TABLE 3.** Circumcised Males From Countries and Cultures in 1980 That Were Not Excluded by Frisch and Simonsen Study

Country	% of Country’s Population That Is Muslim	Total Number of Residents in Denmark	Number of Muslims in Denmark
Countries not excluded having >10% Muslim populations and number of residents in Denmark in 1980			
Bulgaria	19.30	119	23
Cyprus	12.24	100	23
Ethiopia	32.77	110	36
Gambia	90.00	57	51
Ghana	15.89	158	25
India	11.36	1730	197
Israel/Palestine	37.00	623	231
Liberia	14.00	20	3
Libya	99.97	22	22
Malaysia	52.93	176	93
Mauritania	100.0	12	12
Mauritius	12.95	41	5
Mozambique	17.65	73	13
Nigeria	47.20	96	45
Senegal	89.69	16	14
Sierra Leone	75.00	19	14
Singapore	16.34	147	24
Sudan	80.10	79	63
Tanzania	30.68	160	49
Uganda	10.55	162	17
Former USSR	16.81	2,364	397
Yemen and N. Yemen	99.98	85	85
Former Yugoslavia^a^	17.83	7452	882
Total			2,326
European countries and their contributions to Danish immigrants in 1980			
Austria	1.02	1,508	15
Belgium	3.60	534	19
France	4.60	2,130	98
Germany	2.20	26,333	579
Greece	1.66	686	11
Netherlands	2.80	1,901	53
Norway	0.13	13,872	18
Sweden	0.30	15,979	48
Switzerland	0.89	1,325	12
United Kingdom	2.20	7,598	167
Total			1,021

Abbreviation: USSR, United Soviet Socialist Republic.

a Total Muslims from Yugoslavia excludes an estimated 447 Kosovans.

In Table 3, to be conservative, we excluded countries with fewer than 10 citizens in Denmark in 1980 as well as countries with populations in which Muslims represented fewer than 10%. Of the countries included, there was a total of 2,326 Muslim immigrants. As some European countries have very large immigrant communities in Denmark on account of proximity and, in many cases, membership of the European Economic Community or European Free Trade Association, even their modest Muslim populations would contribute a significant proportion of Muslims, assuming their movements are as great as their non-Muslim countryfolk. We also excluded countries that contribute fewer than 10 Muslims (Table 3). This resulted in the identification of a further 1,021 Muslims. Table 4 shows data for non-Muslim cultures that practice early MC and have a significant presence in Denmark. To be conservative, we included only countries in which more than 70% of males are circumcised. The number of non-Muslim immigrants from these countries was 7,592.

**TABLE 4.** Non-Muslim Immigrants in 1980 in Denmark From Countries in Which Prevalence of Male Circumcision Is More Than 70% But That Were Not Excluded by Frisch and Simonsen Study

	**% of Country’s Population That Is Muslim**	**Total Number of Residents in Denmark**	**Number of Non-Muslims in Denmark**
Ethiopia	32.77	110	74
Israel/Palestine	37.0	623	392
Ghana	15.89	158	133
Nigeria	47.2	96	51
Philippines	4.57	909	867
South Korea	0.02	381	381
United States	1.3	5,769	5,694
Total			7,592

Taken together, for 1980, the data suggests a total of 3,347 Muslim immigrants and an additional 7,592 non-Muslim immigrants from countries and cultures that perform MC in childhood but which were not excluded by Frisch and Simonsen. The population of Denmark in 1980 was 5,123,029, of whom 16,394 (0.32%) were Muslim.^136^ The additional 3,347 Muslims we found constitute 0.07% of the Danish population and the 7,592 non-Muslims another 0.15%.

The number of Jews in Denmark is estimated at approximately 7,000 individuals.^140^ Ever since around 2000, it has not been possible to have nontherapeutic circumcision of boys performed at public hospitals in Denmark, as was previously the case. Circumcisions of Jewish boys take place in private homes and are performed by a mohel (traditional Jewish circumciser) under the supervision of a licensed medical doctor in accordance with current Danish rules. In addition to this, an unknown number of circumcisions of boys with a Jewish family background are performed at private medical clinics.

After adding a further 0.2% (approximately 10,000) that were non-Israeli Jews, the total becomes 0.42%. Assuming these people give birth at a similar rate as ethnic Danes (which is likely conservative as immigrants tend to have higher birth rates overall) then their offspring will account for a substantial proportion of the 0.42% of Frisch and Simonsen’s cohort who had undergone non-therapeutic MC. Therefore, our calculations suggest that their cohort likely suffers from substantial residual confounding from ethnicity.

### Failure to Address Biological Plausibility

Frisch and Simonsen did not address the question of biological plausibility (as we discussed earlier).

### Other Issues

The same Danish patient database was included in previous studies by Frisch et al. of sexual function and pleasure,^120^ autism spectrum disorder,^141^ and meatal stenosis.^142^ The percentage of circumcised males in those studies was approximately tenfold higher than their recent study. Problems with statistical analyses, inclusion criteria, and other issues in each study were identified by the present authors^143^^–^^145^ and others.^146^^–^^149^ Additionally, those earlier studies used the same algorithm to control for ethnicity but, as we have shown in this article, that algorithm failed.

Frisch and Simonsen use the term “intact” for uncircumcised males, which implies that circumcised males are missing something important, whereas the commonly used term, “uncircumcised,” is well established in both medical and lay literature.

Examples of obfuscation are also apparent. Instead of referring to negative findings for STIs other than genital herpes as nonsignificant, they refer to them as “inconspicuous.” Another feature is selective citation of outlier studies and opinion pieces but not the rebuttals of each.^150^

Under ideal circumstances, the best study design to address whether early MC reduced STI risk would be a large, well-designed RCT with follow-up of at least 20 years in a setting in which there was a roughly similar proportion of circumcised and uncircumcised men to ensure adequate power. However, such a study would be impractical, costly, and, given the existing biological data showing MC protects against particular STIs in populations in which most men were circumcised in infancy, likely to be deemed unethical by ethics committees.

## CONCLUSIONS

Circumcision early in life has been associated with the reduced risk of HIV and other STIs in men during heterosexual intercourse and during insertive anal intercourse in MSM. In HICs, MSM disproportionately contribute to STI prevalence. This, and lack of protection afforded by MC in men who assume the receptive role during anal intercourse, account for the failure in population studies to detect a role for MC in protecting against STIs in such settings. We illustrated this by more deeply analyzing data from the Canadian study by Nayan et al.^44^

The Frisch and Simonsen study that found early circumcision did not protect against HIV or other STIs^80^ was related to very low case numbers, very low MC prevalence of the Danish cohort studied, failure to consider the issues above of sexual practices among MSM, failure to adequately exclude males with parents from countries in which childhood MC is common, and biological implausibility.

A study in the United States should be best able to test the hypothesis that infant MC protects against STIs later in life. This is because (1) the prevalence of circumcised and uncircumcised males in the United States is similar, (2) most circumcisions in the United States occur in early infancy, (3) the U.S. population size is large, (4) STIs are prevalent in the population, and (5) the United States has a large number of national government health-related datasets.^151^

## Footnotes

* We emailed the trials’ principal investigators (PIs) to determine how many participants were men who have sex with men (MSM). The Kenya trial PI stated that although MSM were not excluded, when participants were asked if they had ever had sex with another man, only 2 responded yes. The Uganda trial PI stated participants weren’t asked about homosexual intercourse in the trial, but our prior studies suggested that this is extremely rare and not culturally accepted in this rural African population.

## Notes

**Peer Reviewed**

**First published online:** August 12, 2022.
